# S1P_3_ Receptor Mediates the Proinflammatory Effect of the Endocannabinoid 2‐Arachidonoylglycerol in Endometriotic Epithelial Cells

**DOI:** 10.1096/fj.202502415R

**Published:** 2025-11-26

**Authors:** Maryam Raeispour, Matteo Prisinzano, Isabelle Seidita, Lucia Romeo, Eleonora Nardi, Francesca Castiglione, Paola Bruni, Felice Petraglia, Caterina Bernacchioni, Chiara Donati

**Affiliations:** ^1^ Department of Experimental and Clinical Biomedical Sciences “M. Serio” University of Florence Florence Italy; ^2^ Histopathology and Molecular Diagnostics Careggi University Hospital Florence Italy

**Keywords:** 2‐arachidonoylglycerol, endocannabinoid system, endometriosis, inflammation, S1P receptors, sphingosine 1‐phosphate

## Abstract

Endometriosis is a chronic inflammatory disease characterized by the ectopic implantation of endometrium outside the uterus associated with pelvic pain and infertility. The molecular mechanisms involved in the pathogenesis of endometriosis are complex and far from being fully elucidated. We recently showed that the signaling of the bioactive sphingolipid sphingosine 1‐phosphate (S1P) is deeply dysregulated in endometriosis. The endocannabinoids anandamide (AEA) and 2‐arachidonoylglycerol (2‐AG), via ligation to G‐protein coupled receptors, CB1, CB2, and GPR18 as well as the cation channel TRPV1, play a crucial role in the modulation of pain and inflammation. Here, the role of endocannabinoid signaling in endometriosis and its possible cross talk with the S1P signaling axis has been investigated. It has been found that CB1, CB2, GPR18, TRPV1 as well as the enzymes involved in endocannabinoid metabolism are expressed in endometriotic lesions. Furthermore, the effect of 2‐AG and AEA in the modulation of inflammation has been established in human endometriotic epithelial cells. 2‐AG, but not methanandamide (MAEA), the nonhydrolyzable AEA analogue, induced a marked increase in the expression of cyclooxygenase 2 and various pro‐inflammatory interleukins (IL‐1β, IL‐6 and IL‐8). Interestingly, S1P_3_, whose expression is augmented by 2‐AG, is crucial for transducing the biological action of the endocannabinoid. Indeed, S1P_3_ pharmacological blockade or its specific silencing impaired the pro‐inflammatory action of 2‐AG. In conclusion, these findings demonstrate, for the first time, the occurrence of a functional interplay between endocannabinoids and S1P signaling in endometriosis, paving the way for novel pharmacological strategies to treat the disease.

## Graphical abstract

The endocannabinoid 2‐arachidonoylglycerol (2‐AG) induced a marked inflammatory response in human endometriotic epithelial cells. A functional cross talk between the signaling pathways of 2‐AG and the bioactive sphingolipid sphingosine 1‐phosphate (S1P) has been identified here in endometriosis. Indeed, the specific S1P receptor S1P_3_, whose expression is augmented by 2‐AG, is crucial for transducing the biological action of the endocannabinoid. Notably, the functional cross talk was found to be independent of both isoforms of the S1P biosynthetic enzyme, sphingosine kinase (SK1/SK2).

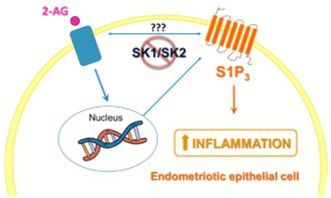

## 1 Introduction

The endocannabinoid system (ECS) has emerged as an important regulator of pain, neuromodulation, and inflammation [1, 2]. The ECS is composed of the endocannabinoids anandamide (AEA) and 2‐arachidonoylglycerol (2‐AG), the cannabinoid G‐protein‐coupled receptors (CB1, CB2, GPR18, and GPR55) and the transient receptor potential cation channel subfamily V member 1 (TRPV1) [3]. In addition, the enzymes responsible for endocannabinoid metabolism such as the biosynthetic enzymes diacylglycerol‐lipase (DAGL) and N‐acylphosphatidyl ethanolamines‐phospholipase D (NAPE‐PLD), as well as the catabolic enzymes fatty acid amide hydrolase (FAAH) and monoacylglycerol lipase (MAGL) are components of ECS. Cannabinoid receptors are expressed in several tissues including the reproductive organs [4, 5]. Interestingly, AEA and 2‐AG are elevated in the plasma of endometriosis‐affected women and increased peritoneal fluid levels of 2‐AG positively correlated with prostaglandin E2 (PGE2), suggesting a possible link between ECS and inflammatory pain [6]. Endometriosis is a chronic benign gynecological endocrine disease characterized by fatigue, heavy menstrual flow, and infertility. The disease, affecting ~10% of women of reproductive age, is defined by the presence of endometrium‐like tissue outside the uterus [7]. Recently, a key role of the bioactive sphingolipid sphingosine 1‐phosphate (S1P) in endometriosis pathogenesis has been solidly demonstrated [8, 9, 10, 11]. Notably, the concentration of S1P is significantly increased in the peritoneal fluid of women with endometriosis [12], and a deep dysregulation of S1P metabolism and signaling has been reported in endometriotic lesions [13, 14].

S1P is involved in the regulation of fundamental cellular and tissue responses such as inflammation, cell survival, migration, and tumorigenesis [15]. S1P synthesis is catalyzed by sphingosine kinases (SK1 and SK2) that phosphorylate sphingosine. The multifaceted effects of S1P mainly rely on the so‐called “inside‐out” mechanism of action, that implies its binding to five specific G‐protein‐coupled receptors, S1P_1–5_, after the export into the extracellular microenvironment mediated by transporters such as Spinster homolog 2 (Spns2) [16, 17].

Current endometriosis treatments comprise surgical and hormonal therapies [18], each linked respectively to high rates of recurrence and side effects in long‐term administration [19, 20]. Thus, the comprehension of the molecular mechanisms implicated in endometriosis pathogenesis might lead to the identification of innovative nonhormonal pharmacological targets for the disease. Although not extensively investigated, literature data support the existence of a functional cross talk between ECS and S1P signaling [21]. For instance, S1P treatment has been shown to modulate CB2 and TRPV1 levels in C2C12 myoblasts [22] and the activation of SK mediates the hypotensive response to AEA in anesthetized mice [23].

The data presented here show for the first time that 2‐AG—but not methanandamide (MAEA), a non‐hydrolyzable AEA analogue—induces a pro‐inflammatory response in endometriotic cells. Our findings also reveal that the S1P‐specific receptor isoform S1P_3_, whose expression is augmented by 2‐AG, is critically involved in the pro‐inflammatory effect of the endocannabinoid. This work provides the first experimental evidence of a functional interplay between endocannabinoids and S1P signaling in endometriosis, pointing at new pharmacological targets for disease treatment.

## 2 Materials and Methods

### 2.1 Materials

All biochemicals, TRI reagent, cell culture reagents, Nutrient Mixture F‐12 Ham (F12), Dulbecco's Modified Eagle Medium (DMEM), fetal bovine serum (FBS), L‐glutamine, Penicillin/Streptomycin, phosphatase inhibitor cocktail, protease inhibitor cocktail, 2‐AG, bovine serum albumin (BSA), VPC23019, TY‐52156, PF‐543, the specific siRNA for SK1, SK2, S1P_1_, S1P_2_, S1P_3_ and the scramble siRNA were purchased from Merck Life Sciences (Burlington, MA, USA). C17 sphingosine and C17‐S1P were obtained from Avanti Polar Lipids (Alabaster, AL, USA).

Human endometriotic epithelial 12Z cell line (Cat. No. T0764) and the Applied Cell Extracellular Matrix were purchased from Applied Biological Materials Inc. (Richmond, BC, Canada). Bradford protein assay, Tris/Glycine/SDS, the EveryBlot Blocking Buffer, the Clarity western ECL substrate and the trans‐blot turbo PVDF membrane were obtained from Bio‐Rad (Hercules, CA, USA). Anti‐S1P_3_ antibody was obtained from Abcam Ltd. (Cambridge, UK). Anti‐SK1, anti‐SK2, anti‐phospho‐SK2 (Thr578), and anti‐phospho‐SK1 (Ser225) antibodies were purchased from ECM Biosciences LLC (Versailles, KY, USA). Anti‐Spns2 antibody was purchased from FabGennix International Inc. Anti‐CB1, anti‐CB2, and Methanandamide (MAEA) were purchased from Cayman Chemical (Ann Arbor, Michigan, USA) and anti‐GPR18 from Invitrogen, Thermo Fisher Scientific (Waltham, MA, USA). Anti‐TRPV1 was obtained from OriGENE Technologies Inc. (Rockville, MD, USA). RayBio C‐Series Human Inflammation Array C1 was from RayBiotech (Peachtree Corners, Georgia, USA). Anti‐GAPDH antibodies and secondary antibodies conjugated to horseradish peroxidase, were obtained from Santa Cruz Biotechnology (Santa Cruz, CA). AllPrep DNA/RNA FFPE kit was purchased from Qiagen (Hilden, Germany). TaqMan Universal Master Mix II, TaqMan gene expression assays, Lipofectamine RNAiMAX, High‐capacity cDNA reverse transcription kit and the UltraVision LP Detection System HRP Polymer and DAB Plus Chromogen were obtained from Thermo Fisher Scientific Inc. (Waltham, MA, USA).

### 2.2 Sample Collection

The gene expression study (*n* = 20) and the immunohistochemistry (IHC) analysis (*n* = 15) were conducted on endometriotic lesions obtained from an independent cohort of patients. Control endometrial samples (*n* = 15) used for gene expression analysis were acquired using diagnostic hysteroscopy procedures. Clinical and imaging investigations were performed to exclude a diagnosis of endometriosis or other uterine disorders in controls. All the samples were histologically characterized. The diagnostic hysteroscopy or surgical procedure for lesion removal was performed during the proliferative phase and all hormonal treatments had been stopped at least 2–3 months before surgery. The endometrial cycle phase was confirmed by histologic analysis of endometrial biopsies. There were no differences in age, pregnancy and parity between the study and control groups. The study protocol was approved by the Institutional Review Board (number 13742) and all patients provided written informed consent.

### 2.3 Cell Culture and Treatment

Human endometriotic epithelial 12Z cells were cultured in dishes coated with the extracellular matrix using a 1:1 mixture of DMEM:F12, supplemented with 10% FBS, 2 mM L‐glutamine, 100 U/mL penicillin/streptomycin at 37°C in 5% CO_2_. For the experiments, cells were serum‐starved overnight in a medium supplemented with 1 mg/mL fatty acid‐free BSA. When requested, cells were pretreated with pharmacological antagonists or an equivalent volume of vehicle (DMSO) for 45 min prior to treatment with 2‐AG or the corresponding vehicle (ethanol).

### 2.4 Cell Transfection

Endometriotic epithelial 12Z cells were transfected with siRNA duplexes using Lipofectamine RNAiMAX, according to the manufacturer's instructions [24, 25]. siRNAs diluted in the mixture of DMEM:F12 were incubated with Lipofectamine RNAiMAX at room temperature (RT) for 20 min and then added to cells to a final concentration of 50 nM, in DMEM:F12 supplemented with 10% FBS. After 30 h cells were serum‐starved overnight and stimulated with 10 μM 2‐AG.

### 2.5 Quantitative Real Time PCR (qPCR)

Total RNA was extracted from Formalin‐Fixed, Paraffin‐Embedded (FFPE) tissue samples using the AllPrep DNA/RNA FFPE kit and from endometriotic epithelial 12Z cells employing TRI‐reagent following the manufacturer instructions, and reverse‐transcribed using the high‐capacity cDNA reverse transcription kit, as directed by the manufacturer. TaqMan gene expression assays were used to quantify target gene mRNAs in triplicate on a CFX96 Touch qPCR Detection System (Bio‐Rad, Hercules, CA, USA). The target sequences were amplified alongside the reference gene β‐actin [14]. The 2^−ΔCt^ and 2^−ΔΔCt^ methods were used to calculate the relative expression of mRNA [26, 27].

### 2.6 Immunohistochemistry

Tissue samples were fixed with formalin, paraffin embedded and 3 μm sections were suitably sliced from each block and then stained for IHC analysis with specific antibodies against CB1 (1:50), CB2 (1:50), GPR18 (1:50), and TRPV1 (1:50) as previously described [28]. The procedure started with deparaffinization and rehydration. The slides were heated in a microwave at 95°C–97°C in a citrate buffer (pH 6.0) for a total of 20 min and cooled to RT for antigen retrieval. Then the slides were incubated with the primary antibodies at 4°C overnight. To detect the antigen–antibody complexes the “UltraVision LP Detection System HRP Polymer and DAB Plus Cromogen” kit was used according to the manufacturer's instructions: tissue slices were covered with the “Primary Antibody Enhancer” for 10 min at RT followed by 15 min with the secondary antibody conjugated to horseradish peroxidase. The bound antibody complexes were stained for 3 min 40 s with diaminobenzidine and then mounted. The slides were analyzed at the Histopathology and Molecular Diagnostics of Careggi University Hospital, Florence. Images were obtained by Optikam PRO6 Digital Camera (C‐P6), OPTIKA microscope Italy, B‐383PLi.

### 2.7 Western Blot (WB) Analysis

Cells were collected in 50 mM Tris, pH 7.5, 120 mM NaCl, 6 mM EGTA, 1 mM EDTA, 20 mM NaF, 15 mM Na_4_P_2_O_7_, 1% Nonidet, with the addition of protease and phosphatase inhibitor cocktail and then incubated at 4°C for 30 min. Cells were centrifuged for 10 000 g, 15 min at 4°C, and the supernatant containing protein was collected. Proteins were used to perform SDS/PAGE and WB. PDVF membranes were incubated at 4°C overnight with the primary antibodies and then with specific secondary antibodies at RT for 1 h. The binding of the antibodies with the specific proteins was detected by chemiluminescence employing Amersham Imager 600 (GE Healthcare, Buckinghamshire, UK).

### 2.8 Human Inflammation Array

Pro‐inflammatory cytokine secretion into the media was quantified by RayBio C‐Series Human Inflammation Array C1. Endometriotic epithelial cells were cultured and treated with 2‐AG or MAEA for 24 h before conditioned media were collected according to the manufacturer's instructions. After the incubation of the media with the antibody arrays' membranes, chemiluminescence signals were obtained employing Amersham Imager 600. Signal intensities were used to quantify inflammatory factors' secretion using ImageJ software.

### 2.9 Sphingosine Kinase in Cell Assay

Endometriotic epithelial cells were plated and upon reaching 70% confluence, were serum‐starved overnight. Cells were incubated with the exogenous substrate C17 sphingosine (5 μM) and contemporaneously stimulated or not with 10 μM 2‐AG for 30 min. Cells were then harvested in methanol and subsequently added to internal standard for C17‐S1P (10 pmol d17 S1P in methanol). Samples were vortexed, precipitated overnight at −80°C, followed by a 5 min centrifuge at 21 300 *g*, 4°C and then the supernatant was analyzed by liquid chromatography–tandem mass spectrometry (LC–MS/MS).

### 2.10 Statistical Analysis

Statistical analysis was performed with the use of Student's *t*‐test, Mann–Whitney test, one‐way or two‐way ANOVA followed by Bonferroni post hoc test. Graphical representations were generated using GraphPad Prism 10.0 (GraphPad Software) (San Diego, CA, USA).

## 3 Results

**FIGURE 1:**
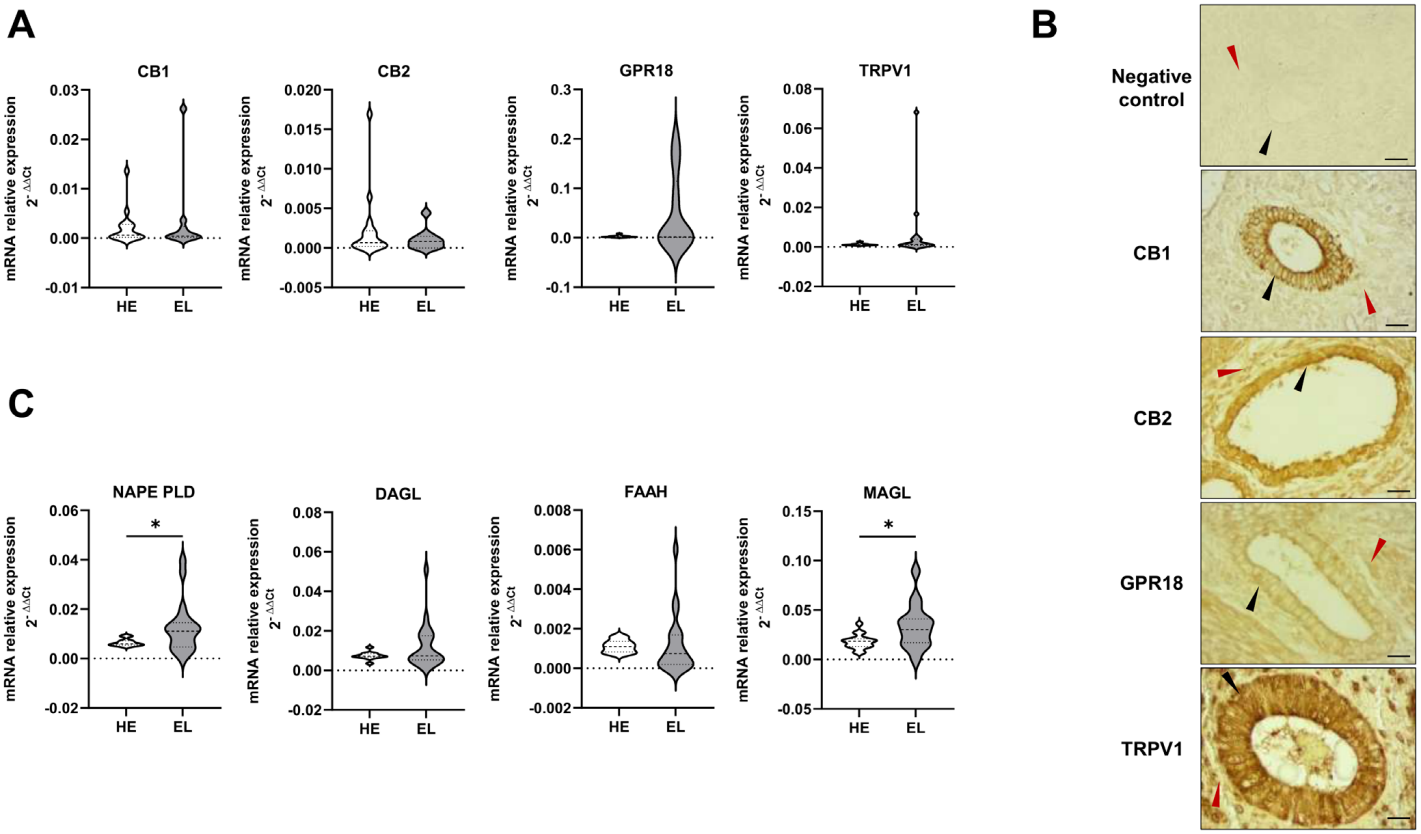
Cannabinoid receptors and enzymes are expressed in human endometriotic lesions. (A) qPCR analysis was performed using TaqMan Gene Expression Assay probes specific for cannabinoid receptors CB1, CB2, GPR18, and TRPV1 in the endometrium of healthy women (HE) (*n* = 15) or endometriotic lesions (EL) of different localization (*n* = 20: 7 ovarian endometriosis and 13 deep infiltrating endometriosis). Results were analyzed employing the 2^−ΔCt^ method. (B) Representative immunohistochemical images of CB1, CB2, GPR18 and TRPV1 expression in endometriotic lesions (*n* = 15: 11 ovarian endometriosis and 4 deep infiltrating endometriosis). The staining with DAB produced a brown precipitate at the site of antibody binding, localized both in epithelial (black arrows) and stromal (red arrows) cells of the endometriotic lesions. The negative control was obtained by processing tissue sections in parallel with the same IHC protocol, omitting the primary antibody. Magnification: ×40, scale bar: 30 μm. (C) qPCR analysis was performed using TaqMan Gene Expression Assay probes specific for cannabinoid enzymes NAPE‐PLD, DAGL, FAAH, and MAGL in the endometrium of healthy women (HE) (*n* = 15) or endometriotic lesions (EL) of different localization (*n* = 20: 7 ovarian endometriosis and 13 deep infiltrating endometriosis). Results were analyzed employing the 2^−ΔCt^ method. NAPE‐PLD and MAGL mRNA levels are significantly increased in EL compared to HE (Student's *t*‐test *p < 0.05).

We initially determined the expression of CB1, CB2, GPR18, and TRPV1 in endometriotic lesions of different localizations. The real‐time PCR analysis reported in Figure 1A shows that all the investigated receptors are expressed at mRNA level in the analyzed lesions. Moreover, the expression levels of each receptor were comparable to those observed in endometrial samples from healthy women (Figure 1A). The expression of CB1, CB2, GPR18, and TRPV1 was confirmed at the protein level, as demonstrated by the representative IHC images shown in Figure 1B: the staining of the analyzed receptors is clearly detectable both in the stroma and in the glands. For all the analyzed receptors, strong immunoreactivity was observed in the glandular epithelium (black arrows), while the stromal cells showed lower signal (red arrows). In addition, the expression of the enzymes involved in 2‐AG and AEA synthesis, NAPE‐PLD and DAGL respectively, as well as in endocannabinoid catabolism, FAAH and MAGL that degrade 2‐AG and AEA, respectively, was demonstrated in endometriotic lesions (Figure 1C). In particular, mRNA levels of NAPE‐PLD and MAGL were found to be significantly increased in endometriotic lesions compared to healthy endometrial tissue (Figure 1C).

In order to characterize the biological action of endocannabinoids in endometriosis, epithelial endometriotic 12Z cells, were employed. We first investigated whether the cells express the endocannabinoid metabolic enzymes and the receptors. qPCR analysis showed that both the biosynthetic (DAGL and NAPE‐PLD) and the catabolic (FAAH and MAGL) enzymes were expressed in endometriotic epithelial cells (Figure S1A). Moreover, the expression of the receptors CB1, CB2, GPR18 and TRPV1 was observed at the mRNA level (Figure S1A) and further confirmed at the protein level by WB analysis (Figure S1B).

**FIGURE 2:**
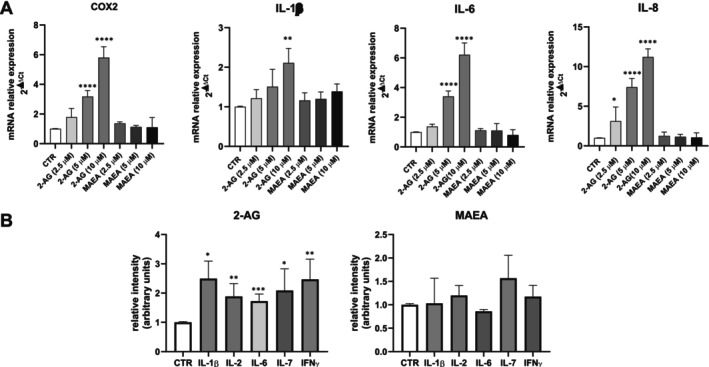
2‐AG, but not MAEA, increases the expression and release of different pro‐inflammatory factors in endometriotic epithelial cells. (A) Endometriotic epithelial cells were serum‐starved for 18 h and treated with increasing concentrations of 2‐AG and MAEA for 24 h. mRNA quantitative analysis of COX2, IL‐1β, IL‐6 and IL‐8 was performed by qPCR. Results, analyzed with the 2^−ΔΔCt^ method, were obtained using β‐Actin as a housekeeping gene and individual inflammatory factors of the unchallenged specimen as a reference gene. 2‐AG increases COX2, IL‐1β, IL‐6 and IL‐8 expression in a statistically significant manner (One‐way ANOVA, *p < 0.05; **p < 0.01; ****p < 0.0001). (B) Endometriotic epithelial cells were serum‐starved for 18 h and treated with 10 μM 2‐AG or 2.5 μM MAEA for 24 h. The obtained conditioned media were screened for the content of proinflammatory markers using the Human Inflammation Array as described in Section 2. Results were expressed as fold increase in respect to control. 2‐AG induces the extracellular release of IL‐1β, IL‐2, IL‐6, IL‐7 and IFNγ in a statistically significant manner (Student's *t*‐test *p < 0.05; **p < 0.01; ***p < 0.001).

Then, we evaluated whether endocannabinoids are able to regulate the inflammatory response in endometriotic epithelial cells. To this aim, the mRNA levels of cyclooxygenase 2 (COX2), interleukin‐1β (IL‐1β), IL‐6 and IL‐8 were examined in the cells treated for 24 h with increasing concentrations (2.5 μM, 5 μM, 10 μM) of 2‐AG and MAEA, the nonhydrolyzable anandamide analogue. By real‐time PCR analysis, it was found that 2‐AG potently increased the expression of COX2, IL‐1β, IL‐6 and IL‐8 with a maximal effect at 10 μM (Figure 2A). On the contrary, the treatment with MAEA did not significantly affect the expression of the analyzed inflammatory factors at any of the tested concentrations (Figure 2A).

In order to confirm these data, we then examined whether the treatment with the endocannabinoids modulates the extracellular release of functional pro‐inflammatory factors. To this aim, the conditioned media obtained from endometriotic cells treated for 24 h with 2‐AG or MAEA were analyzed employing an antibody array that allows the simultaneous detection of multiple cytokines. In agreement with the results obtained by real‐time PCR, 2‐AG significantly increased the extracellular release of various pro‐inflammatory cytokines, namely IL‐1β, IL‐2, IL‐6, IL‐7 and IFNγ (Figure 2B). In contrast, MAEA treatment did not significantly affect the extracellular release of these inflammatory factors in 12Z cells (Figure 2B).

**FIGURE 3:**
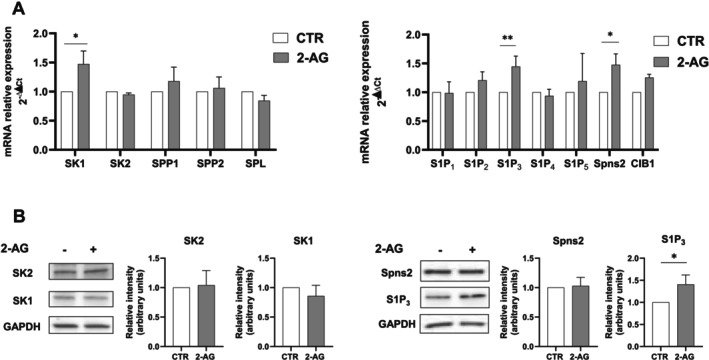
2‐AG modulates S1P signaling axis in endometriotic epithelial cells. Endometriotic epithelial cells were serum‐starved for 18 h and treated with 10 μM 2‐AG for 24 h. (A) mRNA quantitative analysis of S1P metabolic enzymes (SK1, SK2, SPP1, SPP2 and SPL) as well as molecules implicated in S1P signaling (S1P_1–5_, the specific transporter Spns2 and the SK1‐activating protein CIB1) was performed by qPCR. Results, analyzed with the 2^−ΔΔCt^ method, were obtained using β‐Actin as a housekeeping gene and individual targets of the unchallenged specimen as a reference gene. 2‐AG increases SK1, S1P_3_, and Spns2 mRNA levels in a statistically significant manner (Student's *t*‐test *p < 0.05; **p < 0.01). (B) Protein lysates were analyzed using SDS‐PAGE electrophoresis and WB, using specific anti‐SK1, anti‐SK2, anti‐S1P_3_, anti‐Spns2 and anti‐GAPDH antibodies. The histograms represent the densitometric analysis of 4 independent experiments. Data are the mean ± SD and are reported as band intensity normalized to the expression of GAPDH, fold change over control (set as 1). 2‐AG increases S1P_3_ protein content in a statistically significant manner (Student's *t*‐test *p < 0.05).

Given the key role of S1P in modulating the inflammatory process in endometrial cells [29], we next examined whether cell treatment with 10 μM 2‐AG for 24 h regulated the expression of molecules involved in S1P metabolism and signaling. Data shown in Figure 3A show that 2‐AG significantly augmented the mRNA levels of SK1, of the receptor isoform S1P_3_ and of the specific transporter Spns2, suggesting the occurrence of an interplay between 2‐AG and S1P signaling in endometriotic cells. In accordance, WB analysis performed in cells treated with 10 μM 2‐AG for 24 h showed that S1P_3_ protein levels were significantly increased by the endocannabinoid (Figure 3B). On the contrary, the 2‐AG‐induced increase in mRNA levels was not mirrored by a parallel increase in the protein content of SK1 and Spns2, at least at the examined time point (Figure 3B). Of note, our group previously reported a significant upregulation of S1P_3_ both at the mRNA and protein levels in endometriotic lesions compared to endometrium from healthy controls [10, 14]. Interestingly, 2‐AG was responsible for a significant increase in CB1 mRNA levels while the expression levels of the other endocannabinoid receptors were not affected (Figure S2).

**FIGURE 4:**
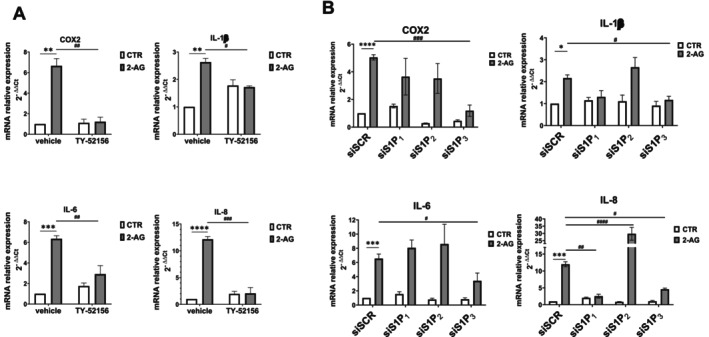
2‐AG pro‐inflammatory action relies on S1P_3_. (A) Serum‐starved endometriotic epithelial cells were pretreated or not with the S1P_3_ antagonist TY‐52156 (10 μM) for 45 min before being challenged with 10 μM 2‐AG for 24 h. mRNA quantitative analysis of COX2, IL‐1β, IL‐6, and IL‐8 was performed by qPCR. Results, analyzed with the 2^−ΔΔCt^ method, were obtained using β‐Actin as a housekeeping gene and individual inflammatory factors of the unchallenged specimen as a reference gene. The blockade of S1P_3_ on 2‐AG‐induced inflammatory effect (**p < 0.01; ***p < 0.001, ****p < 0.0001) was statistically significant by two‐way ANOVA followed by Bonferroni's post hoc test (^#^ *p* < 0.05; ^##^ *p* < 0.01; ^###^ *p* < 0.001). (B) Endometriotic epithelial cells transfected with SCR‐, S1P_1_‐, S1P_2_‐ and S1P_3_‐siRNA were serum‐starved prior to being challenged with 10 μM 2‐AG for 24 h. mRNA quantitative analysis of COX2, IL‐1β, IL‐6 and IL‐8 was performed by qPCR. Results, analyzed with the 2^−ΔΔCt^ method, were obtained using β‐Actin as a housekeeping gene and individual inflammatory factors of the unchallenged specimen as a reference gene. The effect of S1P_3_ downregulation in the reduction of 2‐AG‐induced inflammatory effect (*p < 0.05; ***p < 0.001; ****p < 0.0001) was statistically significant by two‐way ANOVA followed by Bonferroni's post hoc test (^#^ *p* < 0.05; ^##^ *p* < 0.01; ^###^ *p* < 0.001; ^####^ *p* < 0.0001).

Next, the potential role of 2‐AG‐dependent upregulation of S1P_3_ in the pro‐inflammatory action of the endocannabinoid was examined. For this purpose, the expression of the pro‐inflammatory factors was evaluated in endometriotic cells challenged with 2‐AG in the presence or absence of TY‐52156 (10 μM), S1P_3_ specific antagonist, or VPC23019 (10 μM), pharmacological antagonist of S1P_1_/S1P_3_. The obtained results show that the enhanced expression of COX2, IL‐1β, IL‐6 and IL‐8 elicited by 2‐AG was significantly reduced by preincubation with TY‐52156 (Figure 4A) or VPC23019 (Figure S3), suggesting a crucial role for S1P_3_ in transmitting the pro‐inflammatory action of 2‐AG.

In order to confirm these findings, the RNA interference approach was employed to efficaciously reduce the expression levels of S1P_1_, S1P_2_ or S1P_3_ (Figure S4A). Notably, the selective knockdown of S1P_3_ impaired the 2‐AG‐induced increase of COX2, IL‐1β and IL‐6 transcription while the downregulation of the other two receptor isoforms did not alter 2‐AG action (Figure 4B). Interestingly, the selective knockdown of S1P_1_ and S1P_3_ significantly reduced the increase of IL‐8 levels elicited by 2‐AG, whereas the silencing of S1P_2_ enhanced the endocannabinoid effect, suggesting positive and negative roles of these receptor isoforms in the specific modulation of IL‐8 expression (Figure 4B).

**FIGURE 5:**
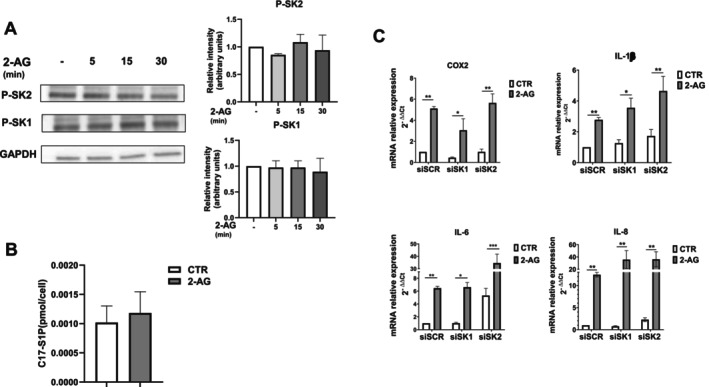
2‐AG pro‐inflammatory action is SK‐independent in endometriotic epithelial cells. (A) Serum‐starved endometriotic epithelial cells were treated with 10 μM 2‐AG for different time intervals (5, 15 and 30 min). Protein lysates were analyzed using SDS‐PAGE electrophoresis and WB, using specific anti‐phospho‐SK1 (P‐SK1), anti‐phospho‐SK2 (P‐SK2) and anti‐GAPDH antibodies. The histograms represent the densitometric analysis of three independent experiments. Data are the mean ± SD and are reported as band intensity normalized to the expression of GAPDH, fold change over control (set as 1). (B) Serum‐starved endometriotic epithelial cells were treated for 30 min with 10 μM 2‐AG before being harvested and then subjected to C17‐S1P quantification by LC–MS/MS as described in the Section 2. Results are the mean ± SEM of three independent experiments and are reported as pmol of S1P normalized on cell number. (C) Endometriotic epithelial cells transfected with SCR‐, SK1‐ and SK2‐siRNA were serum‐starved prior to being challenged with 10 μM 2‐AG for 24 h. mRNA quantitative analysis of COX2, IL‐1β, IL‐6 and IL‐8 was performed by qPCR. Results, analyzed with the 2^−ΔΔCt^ method, were obtained using β‐Actin as a housekeeping gene and individual inflammatory factors of the unchallenged specimen as a reference gene. The effect of SK1 or SK2 downregulation did not significantly affect the 2‐AG inflammatory effect (*p < 0.05; **p < 0.01; ***p < 0.001).

To gain further insight into the mechanism by which 2‐AG exploits S1P signaling to exert its pro‐inflammatory effect, we extensively examined the possible involvement of SKs, the enzymes responsible for S1P synthesis. Indeed, on the basis of the S1P “inside‐out” mechanism of action, the sphingolipid generated following SK activation acts as a ligand of its receptors after being exported outside the cells [16, 17]. Since 2‐AG treatment was unable to enhance SK protein content (Figure 3B), we examined whether the short‐term regulation of SK1 and SK2 via their phosphorylation [30, 31] was implicated in the endocannabinoid action. Data obtained by WB analysis using specific anti‐phospho‐SK1 or anti‐phospho‐SK2 antibodies showed that cell challenge with 10 μM 2‐AG for different time intervals (5 to 30 min) did not affect SK1 or SK2 phosphorylation, ruling out SK1/SK2 activation by the endocannabinoid (Figure 5A). In agreement, the cell assay of sphingosine kinase activity further excluded the activation of SK after 2‐AG treatment (Figure 5B). Indeed, the intracellular levels of S1P quantified by LC–MS/MS were not affected by the treatment with 2‐AG (Figure 5B). Moreover, in order to further verify the involvement of SK in mediating the 2‐AG effect, SK1 and SK2 were efficaciously knocked down by employing siRNA technology (Figure S4B). As depicted in Figure 5C, the 2‐AG‐induced expression of COX2, IL‐1β, IL‐6 and IL‐8 was unaffected by the downregulation of SK1 or SK2, thus demonstrating that the pro‐inflammatory action of 2‐AG did not depend on either SK1 or SK2 in endometriotic epithelial cells.

In conclusion, our findings support the view that the specific S1P_3_ isoform transduces the pro‐inflammatory action of 2‐AG in endometriotic epithelial cells and establishes the rationale for the exploitation of S1P_3_ targeting as an innovative nonhormonal approach to counteract endometriosis.

## 4 Discussion

Endometriosis is a chronic disease with life‐impacting symptoms and high prevalence in women of reproductive age [32]. Inflammation is a cardinal feature of endometriosis, strongly linked with its pathogenesis and the development of symptoms [33].

The cannabinoid receptors have long been known to play a role in inflammatory regulation through both pro‐inflammatory and anti‐inflammatory mechanisms, depending on the cellular environment and pathological state [34]. Here, CB1, CB2, GPR18 and TRPV1 were found to be expressed in healthy endometrium and endometriotic lesions. The expression of CB1, CB2 and TRPV1 has been previously reported in the endometrium and endometriotic lesions [35, 36, 37, 38, 39], while this study represents the first experimental evidence as regards the expression of the receptor GPR18 in endometriosis. Our findings are in accordance with a previous work by Sanchez and coworkers [40] showing that CB1, CB2 and TRPV1 transcript levels were not different between endometrial stromal cells from endometriosis‐affected women and healthy controls in the proliferative phase. Conversely, a significant increase in TRPV1 mRNA and protein levels was observed in rectosigmoid deep infiltrating endometriosis nodules [41]. Based on immunohistochemical analyses, endocannabinoid receptors CB1 and CB2 were found to be downregulated in endometriotic tissues [42]. Analogously, in accordance with the literature [39, 40, 42] we here demonstrate the expression of the endocannabinoid metabolic enzymes NAPE‐PLD, DAGL, FAAH and MAGL in healthy endometrium and endometriotic lesions. Limited literature data are available as regards alterations in the expression of endocannabinoid‐metabolizing enzymes in endometriotic lesions in respect to controls. In particular, it was shown that FAAH and NAPE‐PLD transcripts did not significantly differ between endometrial stromal cells from endometriosis‐affected women and healthy controls [40] whereas NAPE‐PLD, DAGL, FAAH and MAGL protein levels were reduced in endometriotic lesions [42]. It will be interesting to analyze, in future studies, whether the expression of ECS components varies across endometriotic lesions of different anatomical localizations, with the aim of correlating the dysregulation of the ECS system to disease phenotype and severity.

In this study 2‐AG, but not the non‐hydrolyzable anandamide analogue, MAEA, was found to strongly upregulate mRNA levels of COX2, enzyme responsible for the conversion of arachidonic acid into prostanoids including prostaglandins, prostacyclin and thromboxane, all lipid mediators critically involved in the regulation of inflammation and pain perception [43]. Moreover, 2‐AG augmented the release of pro‐inflammatory cytokines IL‐1β, IL‐6 and IL‐8 in endometriotic epithelial cells, confirming the role of ECS in the modulation of inflammation. Differential biological roles of endocannabinoid species have been previously extensively reported. For instance, 2‐AG, but not AEA, exerted neuroprotective effects on granule cells in the dentate gyrus of the hippocampus [44], while only AEA inhibited amyloid β aggregation [45].

In accordance with the here reported proinflammatory role of 2‐AG in endometriotic cells, increased levels of the endocannabinoid in the peritoneal fluid of women affected by endometriosis positively correlated with PGE2 concentration, a key mediator of inflammation [6]. Moreover, in endometrial inflammation the selective activation of CB2 was demonstrated to be associated with the release of nitric oxide, a key player in immune regulation and inflammation [46]. 2‐AG has also been reported to increase the production of chemokines in HL‐60 cells [47], while its seminal plasma levels are higher in the presence of inflammation [48]. However, in animal models of endometriosis, treatment with the two main constituents of the plant *Cannabis sativa* able to bind the cannabinoid receptors, D9‐tetrahydrocannabinol and cannabidiol, was shown to ameliorate the endometriosis‐associated pain [49] and inflammation [50], respectively, pointing at differential effects exerted by phytocannabinoids and endocannabinoids. It is worth noting that both AEA and 2‐AG exert anti‐inflammatory effects reducing the production of pro‐inflammatory cytokines in immune cells [51, 52, 53]. In addition, 2‐AG decreased the expression of COX2 elicited by different inflammatory stimuli in hippocampal neurons [54, 55] and leptin‐induced reactive oxygen species formation in hypothalamic neurons [56].

Here, an interplay between ECS and S1P signaling in the production of inflammatory factors has been revealed in endometriosis. Indeed, 2‐AG was found to modulate the S1P signaling axis since the treatment with the endocannabinoid upregulated the mRNA and protein content of S1P_3_, while it augmented SK1 and Spns2 only at the transcriptional level. The reported findings showing S1P signaling modulation by ECS are in line with a previous study by Standoli et al. [57] in which it was demonstrated that pharmacological stimulation of CB2 counteracted the LPS‐induced increase of SK1 and SK2 transcription in microglial cells. Reciprocally, ECS signaling regulation by S1P has been shown since the sphingolipid augmented the expression of TRPV1, while it reduced CB2 levels in skeletal muscle cells [22]. Interestingly, here S1P_3_ was demonstrated to mediate the pro‐inflammatory action of 2‐AG since its pharmacological inhibition or downregulation by gene silencing significantly reduced the elevated expression of COX‐2, IL‐1β, IL‐6 and IL‐8 elicited by the endocannabinoid. These results provide compelling evidence that the upregulation of the analyzed pro‐inflammatory molecules by 2‐AG relies on S1P_3_ engagement. However, this does not exclude that, in a reciprocal manner, the 2‐AG–induced upregulation of S1P_3_ is at least in part mediated by pro‐inflammatory factors, thereby supporting a positive feedback mechanism that sustains inflammation. Notably, our research group has already provided multiple pieces of evidence that S1P_3_ is crucially involved in endometriosis pathogenesis. Indeed, this receptor subtype has been found to be upregulated in endometriotic lesions [10, 14] and to positively correlate with the fibrosis extent of the disease [10]. Moreover, S1P_3_ mediated the pro‐fibrotic action of S1P [10] as well as the pro‐invasive phenotype elicited by neuropeptide S [9] in endometriotic epithelial cells. Notably, in relation to the regulation of IL‐8 levels, the data presented in this study demonstrated not only the involvement of S1P_3_, similarly to what was observed for the other cytokines, but also a further positive role for S1P_1_ and a negative regulatory role for S1P_2_. Indeed, when this latter receptor isoform was silenced in endometriotic epithelial cells, IL‐8 levels were significantly increased following treatment with 2‐AG, compared to control cells. This role of S1P_2_ contrasts with previous findings in extravillous trophoblast‐derived HTR‐8/SVneo cells, where IL‐8 release was dependent on the S1P_2_/Rho signaling axis, highlighting that such regulatory mechanisms are highly context‐specific [58].

Compelling evidence from the literature indicates that various extracellular cues elicit their biological effects through the regulation of SK1 and SK2, causing enhanced production of S1P, that after its export outside the cell can engage its cognate receptors S1PR [59]. Crucially here, 2‐AG was demonstrated to be unable to enhance the catalytic activity of SK and consistently, SK‐specific downregulation did not interfere with its pro‐inflammatory action, thus ruling out the involvement of SK/S1P_3_ inside‐out signaling in endometriotic epithelial cells. On the contrary, SK1 activation induced by the endocannabinoid AEA was required to recruit S1P_3_ and mediate vasorelaxation in the rat coronary artery [60] and the SK1/S1P regulatory axis was necessary for the rapid hypotension induced by AEA in anesthetized mice [23].

The action of 2‐AG likely depends on its interaction with one or more specific receptors, found to be expressed in the endometriotic epithelial cells. However, our preliminary experimental approaches aimed at identifying the cannabinoid receptor involved in mediating the pro‐inflammatory effect of 2‐AG have so far not yielded robust results. Since the data presented here show that SK1/SK2 is not required to mediate the effects of 2‐AG, one possible explanation for the activation of S1P_3_ by 2‐AG is a direct interaction between cannabinoid and S1P_3_ receptors. Further studies are required to clarify the molecular mechanism by which 2‐AG leads to S1P_3_ transactivation in endometriotic cells. Of note, S1PR and CB1/CB2 belong to class A of GPCR and share 20% sequence identity. Notably, S1P_5_ has been reported to exert a negative regulation of the tumorigenic effect induced by CB2 in glioblastoma cells: bioluminescence resonance energy transfer analysis highlighted that this S1P receptor subtype strongly and specifically interacts with CB2 [61].

Collectively, these findings enhance our understanding of the molecular mechanisms involved in the development of endometriosis pathogenesis, highlighting the critical role of S1P_3_ in mediating the pro‐inflammatory action of 2‐AG in endometriotic epithelial cells. S1PR modulators have emerged as promising therapeutics for various immune‐mediated diseases, including multiple sclerosis, inflammatory bowel disease and psoriasis, by modulating lymphocyte trafficking and reducing tissue inflammation [62]. Since we recently demonstrated that S1P_3_ is involved in the fibrotic and invasive traits of endometriotic cells [9, 10], the here presented data further support the potential of targeting S1P_3_ as a novel nonhormonal therapeutic strategy for endometriosis treatment.

## Author Contributions

M.R., M.P., I.S., L.R. and E.N. performed the experiments and contributed to data analysis. P.B., F.P., C.B. and C.D. conceived or designed the experiments. C.B., C.D. and F.C. participated in data analysis. P.B., C.B. and C.D. wrote the manuscript. F.P. revised the paper. All authors read and approved the final manuscript.

## Conflicts of Interest

The authors declare no conflicts of interest.

## Supporting information


**Figures S1‐S4:** fsb271255‐sup‐0001‐FigureS1‐S4.pdf.

## Data Availability

All data generated or analyzed during this study are included in this published article. The analyzed datasets and materials used in the current study are available from the corresponding authors on reasonable request.

## References

[1] H. Lowe , N. Toyang , B. Steele , J. Bryant , and W. Ngwa , “The Endocannabinoid System: A Potential Target for the Treatment of Various Diseases,” International Journal of Molecular Sciences 22 (2021): 9472. 34502379 10.3390/ijms22179472 PMC8430969

[2] H.‐C. Lu and K. Mackie , “Review of the Endocannabinoid System,” Biological Psychiatry: Cognitive Neuroscience and Neuroimaging 6 (2021): 607–615. 32980261 10.1016/j.bpsc.2020.07.016 PMC7855189

[3] N. Joshi and E. S. Onaivi , “Endocannabinoid System Components: Overview and Tissue Distribution,” in Recent Advances in Cannabinoid Physiology and Pathology, vol. 1162, ed. A. N. Bukiya (Springer International Publishing, 2019), 1–12. 10.1007/978-3-030-21737-2_1 31332731

[4] B. Gatta‐Cherifi and D. Cota , “New Insights on the Role of the Endocannabinoid System in the Regulation of Energy Balance,” International Journal of Obesity 40 (2016): 210–219. 26374449 10.1038/ijo.2015.179

[5] N. Dmitrieva , H. Nagabukuro , D. Resuehr , et al., “Endocannabinoid Involvement in Endometriosis,” Pain 151 (2010): 703–710. 20833475 10.1016/j.pain.2010.08.037 PMC2972363

[6] T. Andrieu , A. Chicca , D. Pellegata , et al., “Association of Endocannabinoids With Pain in Endometriosis,” Pain 163 (2022): 193–203. 34001768 10.1097/j.pain.0000000000002333 PMC8675052

[7] C. Chapron , M.‐C. Lafay‐Pillet , P. Santulli , et al., “A New Validated Screening Method for Endometriosis Diagnosis Based on Patient Questionnaires,” eClinicalMedicine 44 (2022): 101263. 35059616 10.1016/j.eclinm.2021.101263 PMC8760436

[8] J. Rudzitis‐Auth , A. Christoffel , M. D. Menger , and M. W. Laschke , “Targeting Sphingosine Kinase‐1 With the Low MW Inhibitor SKI‐5C Suppresses the Development of Endometriotic Lesions in Mice,” British Journal of Pharmacology 178 (2021): 4104–4118. 34185874 10.1111/bph.15601

[9] M. Prisinzano , C. Bernacchioni , I. Seidita , et al., “Sphingosine 1‐Phosphate Signaling Axis Mediates Neuropeptide S‐Induced Invasive Phenotype of Endometriotic Cells,” FEBS Journal 291 (2024): 1744–1758. 38287231 10.1111/febs.17071

[10] C. Bernacchioni , M. Rossi , V. Vannuzzi , et al., “Sphingosine‐1‐Phosphate Receptor 3 Is a Non‐Hormonal Target to Counteract Endometriosis‐Associated Fibrosis,” Fertility and Sterility 121 (2024): 631–641. 38072366 10.1016/j.fertnstert.2023.12.007

[11] M. Prisinzano , I. Seidita , P. Bruni , et al., “Characterization of Functionally Relevant G Protein‐Coupled Receptors in Endometriotic Epithelial Cells,” Cellular Signalling 133 (2025): 111876. 40381972 10.1016/j.cellsig.2025.111876

[12] Y. Ono , T. Kawakita , O. Yoshino , et al., “Sphingosine 1‐Phosphate (S1P) in the Peritoneal Fluid Skews M2 Macrophage and Contributes to the Development of Endometriosis,” Biomedicine 9 (2021): 1519. 10.3390/biomedicines9111519 PMC8614877 34829748

[13] P. Santulli , L. Marcellin , J.‐C. Noël , et al., “Sphingosine Pathway Deregulation in Endometriotic Tissues,” Fertility and Sterility 97 (2012): 904–911. 22277765 10.1016/j.fertnstert.2011.12.051

[14] C. Bernacchioni , T. Capezzuoli , V. Vannuzzi , et al., “Sphingosine 1‐Phosphate Receptors Are Dysregulated in Endometriosis: Possible Implication in Transforming Growth Factor β‐Induced Fibrosis,” Fertility and Sterility 115 (2021): 501–511. 32907751 10.1016/j.fertnstert.2020.08.012

[15] Y. A. Hannun and L. M. Obeid , “Sphingolipids and Their Metabolism in Physiology and Disease,” Nature Reviews. Molecular Cell Biology 19 (2018): 175–191. 29165427 10.1038/nrm.2017.107 PMC5902181

[16] V. A. Blaho and T. Hla , “An Update on the Biology of Sphingosine 1‐Phosphate Receptors,” Journal of Lipid Research 55 (2014): 1596–1608. 24459205 10.1194/jlr.R046300 PMC4109755

[17] T. Nishi , N. Kobayashi , Y. Hisano , A. Kawahara , and A. Yamaguchi , “Molecular and Physiological Functions of Sphingosine 1‐Phosphate Transporters,” Biochimica et Biophysica Acta (BBA)—Molecular and Cell Biology of Lipids 1841 (2014): 759–765. 23921254 10.1016/j.bbalip.2013.07.012

[18] S. Vannuccini , S. Clemenza , M. Rossi , and F. Petraglia , “Hormonal Treatments for Endometriosis: The Endocrine Background,” Reviews in Endocrine & Metabolic Disorders 23 (2022): 333–355. 34405378 10.1007/s11154-021-09666-w PMC9156507

[19] J. Brown , T. J. Crawford , C. Allen , S. Hopewell , and A. Prentice , “Nonsteroidal Anti‐Inflammatory Drugs for Pain in Women With Endometriosis,” Cochrane Database of Systematic Reviews 1 (2017): CD004753. 28114727 10.1002/14651858.CD004753.pub4 PMC6464974

[20] A. Zakhari , E. Delpero , S. McKeown , G. Tomlinson , O. Bougie , and A. Murji , “Endometriosis Recurrence Following Post‐Operative Hormonal Suppression: A Systematic Review and Meta‐Analysis,” Human Reproduction Update 27 (2021): 96–107. 33020832 10.1093/humupd/dmaa033 PMC7781224

[21] C. Rapino , S. Standoli , F. Cencetti , P. Bruni , S. Oddi , and M. Maccarrone , “New Insights Into the Crosstalk Between Endocannabinoids and Sphingosine‐1‐Phosphate,” Journal of Biological Chemistry 301 (2025): 110781. 41033556 10.1016/j.jbc.2025.110781 PMC12597274

[22] S. Standoli , S. Pecchioli , D. Tortolani , et al., “The TRPV1 Receptor Is Up‐Regulated by Sphingosine 1‐Phosphate and Is Implicated in the Anandamide‐Dependent Regulation of Mitochondrial Activity in C2C12 Myoblasts,” International Journal of Molecular Sciences 23 (2022): 11103. 36232401 10.3390/ijms231911103 PMC9570403

[23] F. H. Greig , K. Nather , M. D. Ballantyne , et al., “Requirement for Sphingosine Kinase 1 in Mediating Phase 1 of the Hypotensive Response to Anandamide in the Anaesthetised Mouse,” European Journal of Pharmacology 842 (2019): 1–9. 30359564 10.1016/j.ejphar.2018.10.027 PMC6318480

[24] F. Cencetti , G. Bruno , S. Blescia , C. Bernacchioni , P. Bruni , and C. Donati , “Lysophosphatidic Acid Stimulates Cell Migration of Satellite Cells. A Role for the Sphingosine Kinase/Sphingosine 1‐Phosphate Axis,” FEBS Journal 281 (2014): 4467–4478. 25131845 10.1111/febs.12955

[25] F. Cencetti , C. Bernacchioni , M. Bruno , et al., “Sphingosine 1‐Phosphate‐Mediated Activation of Ezrin‐Radixin‐Moesin Proteins Contributes to Cytoskeletal Remodeling and Changes of Membrane Properties in Epithelial Otic Vesicle Progenitors,” Biochimica et Biophysica Acta (BBA)—Molecular Cell Research 1866 (2019): 554–565. 30611767 10.1016/j.bbamcr.2018.12.007

[26] K. J. Livak and T. D. Schmittgen , “Analysis of Relative Gene Expression Data Using Real‐Time Quantitative PCR and the 2^−ΔΔCT^ Method,” Methods 25 (2001): 402–408. 11846609 10.1006/meth.2001.1262

[27] T. D. Schmittgen and K. J. Livak , “Analyzing Real‐Time PCR Data by the Comparative CT Method,” Nature Protocols 3 (2008): 1101–1108. 18546601 10.1038/nprot.2008.73

[28] M. Prisinzano , M. Raeispour , M. Rossi , et al., “Expression of Cannabinoid Receptors CB1, CB2 and GPR18 in Adenomyotic Lesions,” Journal of Endometriosis and Uterine Disorders 10 (2025): 100111.

[29] I. Seidita , I. Tusa , M. Prisinzano , et al., “Sphingosine 1‐Phosphate Elicits a ROS‐Mediated Proinflammatory Response in Human Endometrial Stromal Cells via ERK5 Activation,” FASEB Journal 37 (2023): e23061. 37389926 10.1096/fj.202300323R

[30] S. M. Pitson , “Activation of Sphingosine Kinase 1 by ERK1/2‐Mediated Phosphorylation,” EMBO Journal 22 (2003): 5491–5500. 14532121 10.1093/emboj/cdg540 PMC213794

[31] N. C. Hait , A. Bellamy , S. Milstien , T. Kordula , and S. Spiegel , “Sphingosine Kinase Type 2 Activation by ERK‐Mediated Phosphorylation,” Journal of Biological Chemistry 282 (2007): 12058–12065. 17311928 10.1074/jbc.M609559200

[32] K. T. Zondervan , C. M. Becker , and S. A. Missmer , “Endometriosis,” New England Journal of Medicine 382 (2020): 1244–1256. 32212520 10.1056/NEJMra1810764

[33] P. T. K. Saunders and A. W. Horne , “Endometriosis: Etiology, Pathobiology, and Therapeutic Prospects,” Cell 184 (2021): 2807–2824. 34048704 10.1016/j.cell.2021.04.041

[34] C. Turcotte , M.‐R. Blanchet , M. Laviolette , and N. Flamand , “The CB2 Receptor and Its Role as a Regulator of Inflammation,” Cellular and Molecular Life Sciences 73 (2016): 4449–4470. 27402121 10.1007/s00018-016-2300-4 PMC5075023

[35] M. Leconte , C. Nicco , C. Ngô , et al., “Antiproliferative Effects of Cannabinoid Agonists on Deep Infiltrating Endometriosis,” American Journal of Pathology 177 (2010): 2963–2970. 21057002 10.2353/ajpath.2010.100375 PMC2993285

[36] A. H. Taylor , M. S. Abbas , M. A. Habiba , and J. C. Konje , “Histomorphometric Evaluation of Cannabinoid Receptor and Anandamide Modulating Enzyme Expression in the Human Endometrium Through the Menstrual Cycle,” Histochemistry and Cell Biology 133 (2010): 557–565. 20369362 10.1007/s00418-010-0695-9

[37] M. Almada , C. Amaral , M. Diniz‐da‐Costa , G. Correia‐da‐Silva , N. A. Teixeira , and B. M. Fonseca , “The Endocannabinoid Anandamide Impairs In Vitro Decidualization of Human Cells,” Reproduction 152 (2016): 351–361. 27568210 10.1530/REP-16-0364

[38] S. Allam , E. Paris , I. Lazcano , et al., “Detection of Cannabinoid Receptor Expression by Endometriotic Lesions in Women With Endometriosis as an Alternative to Opioid‐Based Pain Medication,” Journal of Immunology Research 2022 (2022): 1–9. 10.1155/2022/4323259 PMC9184153 35692500

[39] H. Lingegowda , B. J. Williams , K. G. Spiess , et al., “Role of the Endocannabinoid System in the Pathophysiology of Endometriosis and Therapeutic Implications,” Journal of Cannabis Research 4 (2022): 54. 36207747 10.1186/s42238-022-00163-8 PMC9540712

[40] A. M. Sanchez , R. Cioffi , P. Viganò , et al., “Elevated Systemic Levels of Endocannabinoids and Related Mediators Across the Menstrual Cycle in Women With Endometriosis,” Reproductive Sciences 23 (2016): 1071–1079. 26887427 10.1177/1933719116630414

[41] N. Bohonyi , K. Pohóczky , B. Szalontai , et al., “Local Upregulation of Transient Receptor Potential Ankyrin 1 and Transient Receptor Potential Vanilloid 1 Ion Channels in Rectosigmoid Deep Infiltrating Endometriosis,” Molecular Pain 13 (2017): 174480691770556. 10.1177/1744806917705564 PMC5424991 28478727

[42] E. Bilgic , E. Guzel , S. Kose , et al., “Endocannabinoids Modulate Apoptosis in Endometriosis and Adenomyosis,” Acta Histochemica 119 (2017): 523–532. 28549792 10.1016/j.acthis.2017.05.005

[43] L. S. Simon , “Role and Regulation of Cyclooxygenase‐2 During Inflammation,” American Journal of Medicine 106 (1999): 37S–42S. 10390126 10.1016/s0002-9343(99)00115-1

[44] S. Kreutz , M. Koch , C. Ghadban , H.‐W. Korf , and F. Dehghani , “Cannabinoids and Neuronal Damage: Differential Effects of THC, AEA and 2‐AG on Activated Microglial Cells and Degenerating Neurons in Excitotoxically Lesioned Rat Organotypic Hippocampal Slice Cultures,” Experimental Neurology 203 (2007): 246–257. 17010339 10.1016/j.expneurol.2006.08.010

[45] M. Khavandi , P. P. N. Rao , and M. A. Beazely , “Differential Effects of Endocannabinoids on Amyloid‐Beta Aggregation and Toxicity,” International Journal of Molecular Sciences 24 (2023): 911. 36674424 10.3390/ijms24020911 PMC9861930

[46] T. Iuvone , D. De Filippis , A. Di Spiezio Sardo , et al., “Selective CB2 Up‐Regulation in Women Affected by Endometrial Inflammation,” Journal of Cellular and Molecular Medicine 12 (2008): 661–670. 18419603 10.1111/j.1582-4934.2007.00085.x PMC3822551

[47] T. Sugiura , S. Kishimoto , S. Oka , and M. Gokoh , “Biochemistry, Pharmacology and Physiology of 2‐Arachidonoylglycerol, an Endogenous Cannabinoid Receptor Ligand,” Progress in Lipid Research 45 (2006): 405–446. 16678907 10.1016/j.plipres.2006.03.003

[48] A. Barbonetti , T. Bisogno , N. Battista , et al., “2‐Arachidonoylglycerol Levels Are Increased in Leukocytospermia and Correlate With Seminal Macrophages,” Andrology 5 (2017): 87–94. 27863106 10.1111/andr.12283

[49] A. Escudero‐Lara , J. Argerich , D. Cabañero , and R. Maldonado , “Disease‐Modifying Effects of Natural Δ9‐Tetrahydrocannabinol in Endometriosis‐Associated Pain,” eLife 9 (2020): e50356. 31931958 10.7554/eLife.50356 PMC6977967

[50] S. B. Okten , C. Cetin , O. E. Tok , et al., “Cannabidiol as a Potential Novel Treatment for Endometriosis by Its Anti‐Inflammatory, Antioxidative and Antiangiogenic Effects in an Experimental Rat Model,” Reproductive Biomedicine Online 46 (2023): 865–875. 36997400 10.1016/j.rbmo.2023.01.018

[51] E. V. Berdyshev , E. Boichot , N. Germain , N. Allain , J.‐P. Anger , and V. Lagente , “Influence of Fatty Acid Ethanolamides and Δ9‐Tetrahydrocannabinol on Cytokine and Arachidonate Release by Mononuclear Cells,” European Journal of Pharmacology 330 (1997): 231–240. 9253958 10.1016/s0014-2999(97)01007-8

[52] Y. Chang , S. T. Lee , and W. Lin , “Effects of Cannabinoids on LPS‐Stimulated Inflammatory Mediator Release From Macrophages: Involvement of Eicosanoids,” Journal of Cellular Biochemistry 81 (2001): 715–723. 11329626 10.1002/jcb.1103

[53] M. T. Cencioni , V. Chiurchiù , G. Catanzaro , et al., “Anandamide Suppresses Proliferation and Cytokine Release From Primary Human T‐Lymphocytes Mainly via CB2 Receptors,” PLoS One 5 (2010): e8688. 20098669 10.1371/journal.pone.0008688 PMC2809084

[54] J. Zhang and C. Chen , “Endocannabinoid 2‐Arachidonoylglycerol Protects Neurons by Limiting COX‐2 Elevation,” Journal of Biological Chemistry 283 (2008): 22601–22611. 18534982 10.1074/jbc.M800524200 PMC2504873

[55] X. Chen , J. Zhang , and C. Chen , “Endocannabinoid 2‐Arachidonoylglycerol Protects Neurons Against β‐Amyloid Insults,” Neuroscience 178 (2011): 159–168. 21256197 10.1016/j.neuroscience.2011.01.024 PMC3052737

[56] L. Palomba , C. Silvestri , R. Imperatore , et al., “Negative Regulation of Leptin‐Induced Reactive Oxygen Species (ROS) Formation by Cannabinoid CB1 Receptor Activation in Hypothalamic Neurons,” Journal of Biological Chemistry 290 (2015): 13669–13677. 25869131 10.1074/jbc.M115.646885 PMC4447947

[57] S. Standoli , C. Rapino , C. Di Meo , et al., “Sphingosine Kinases at the Intersection of Pro‐Inflammatory LPS and Anti‐Inflammatory Endocannabinoid Signaling in BV2 Mouse Microglia Cells,” International Journal of Molecular Sciences 24 (2023): 8508. 37239854 10.3390/ijms24108508 PMC10217805

[58] D. Brünnert , S. Piccenini , J. Ehrhardt , M. Zygmunt , and P. Goyal , “Sphingosine 1‐Phosphate Regulates IL‐8 Expression and Secretion via S1PR 1 and S1PR 2 Receptors‐Mediated Signaling in Extravillous Trophoblast Derived HTR‐8/SVneo Cells,” Placenta 36 (2015): 1115–1121. 26321412 10.1016/j.placenta.2015.08.010

[59] S. Spiegel and S. Milstien , “The Outs and the Ins of Sphingosine‐1‐Phosphate in Immunity,” Nature Reviews. Immunology 11 (2011): 403–415. 10.1038/nri2974 PMC3368251 21546914

[60] K. Mair , E. Robinson , K. Kane , et al., “Interaction Between Anandamide and Sphingosine‐1‐Phosphate in Mediating Vasorelaxation in Rat Coronary Artery,” British Journal of Pharmacology 161 (2010): 176–192. 20718749 10.1111/j.1476-5381.2010.00878.x PMC2962826

[61] Y. Kim and S. Ghil , “Negative Regulation of Cannabinoid Receptor 2‐Induced Tumorigenic Effect by Sphingosine‐1‐Phosphate Receptor 5 Activation,” Oncology Reports 53 (2025): 41. 39918009 10.3892/or.2025.8874

[62] M. P. McGinley and J. A. Cohen , “Sphingosine 1‐Phosphate Receptor Modulators in Multiple Sclerosis and Other Conditions,” Lancet 398 (2021): 1184–1194. 34175020 10.1016/S0140-6736(21)00244-0

