# Supplementary material for: S1P3 Receptor Mediates the Proinflammatory Effect of the Endocannabinoid 2‐Arachidonoylglycerol in Endometriotic Epithelial Cells
Source: FASEB J. 2025 Nov 26;39(22):e71255. doi: 10.1096/fj.202502415R (PMC12651104; doi:10.1096/fj.202502415R)
Supplement: Supplementary file 1 — Figures S1‐S4: fsb271255‐sup‐0001‐FigureS1‐S4.pdf. [file FSB2-39-e71255-s001.pdf]

SUPPLEMENTAL INFORMATION

TITLE

S1P$_3$ receptor mediates the proinflammatory effect of the endocannabinoid 2-arachidonoylglycerol in endometriotic epithelial cells.

Authors

Maryam Raeispour*[1], Matteo Prisinzano*[1], Isabelle Seidita[1], Lucia Romeo[1], Eleonora Nardi[2], Francesca Castiglione[2], Paola Bruni[1], Felice Petraglia[1], Caterina Bernacchioni[#1] and Chiara Donati[#1]

[1]Department of Experimental and Clinical Biomedical Sciences "M. Serio," University of Florence Florence, Italy.

[2]Histopathology and Molecular Diagnostics, Careggi University Hospital Florence, Italy

Felice Petraglia, felice.petraglia@unifi.it, corresponding author

***shared first authorship**

[#]shared last authorship

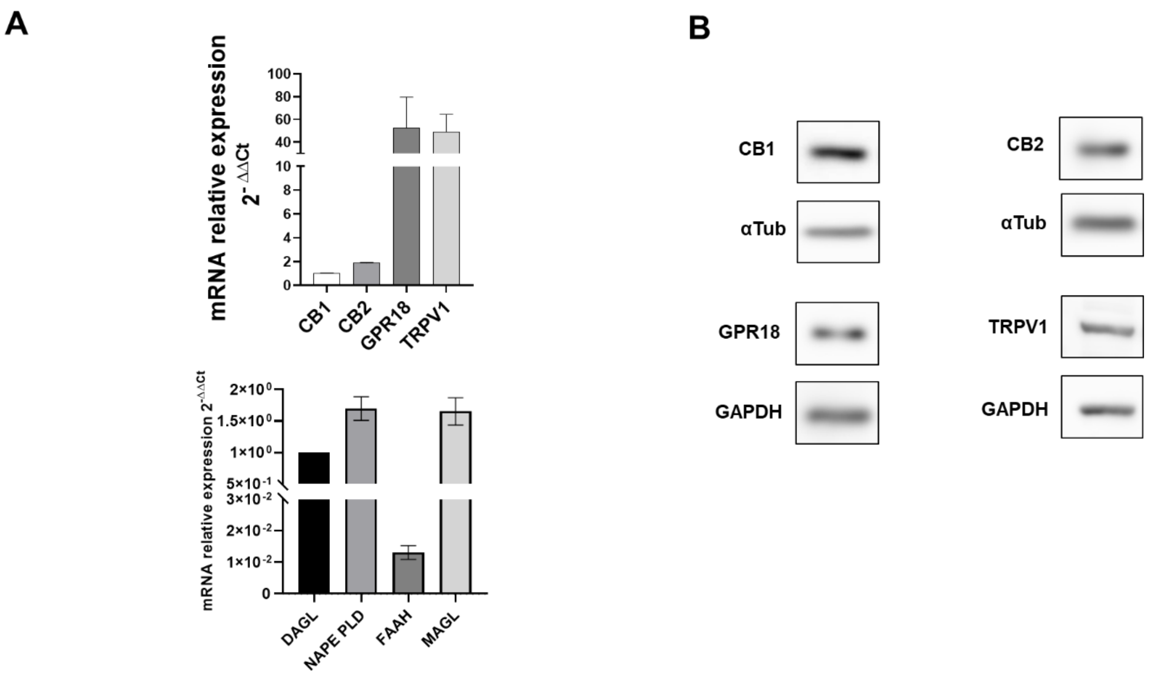

Figure S1: Endometriotic epithelial cells express cannabinoid receptors and enzymes. (A) qPCR analysis was performed using TaqMan Gene Expression Assay probes specific for cannabinoid receptors CB1, CB2, GPR18 and TRPV1 as well as for the enzymes NAPE-PLD, DAGL, FAAH and MAGL in human endometriotic epithelial cells. Results, analyzed with the $2^{-\Delta\Delta Ct}$ method, were obtained using β-Actin as a housekeeping gene and CB1 (upper panel) or DAGL (lower panel) as a reference gene. Data are mean ± SEM of three independent experiments. **(B)** WB analysis was performed using antibodies specific for CB1 (expected molecular weight ~53 kDa), CB2 (expected molecular weight ~40 kDa), GPR18 (expected molecular weight ~38 kDa) and TRPV1 (expected molecular weight ~95 kDa) in human endometriotic epithelial cells.

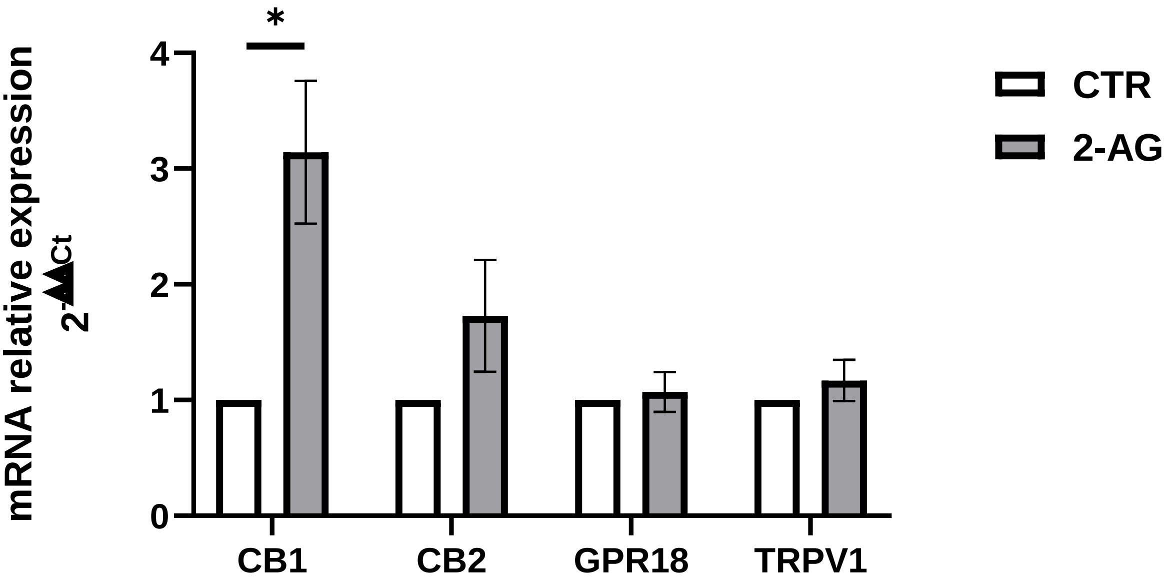

Figure S2: Effect of 2-AG on endocannabinoid receptor expression. qPCR analysis was performed using TaqMan Gene Expression Assay probes specific for cannabinoid receptors CB1, CB2, GPR18 and TRPV1 in human endometriotic epithelial 12Z cells treated or not with 10 µM 2-AG for 24 h. Results, analyzed with the $2^{-\Delta\Delta Ct}$ method, were obtained using β-Actin as a housekeeping gene and individual targets of the unchallenged specimen as a reference gene. 2-AG increases CB1 mRNA levels in a statistically significant manner (Student's t-test $*p < 0.05$).

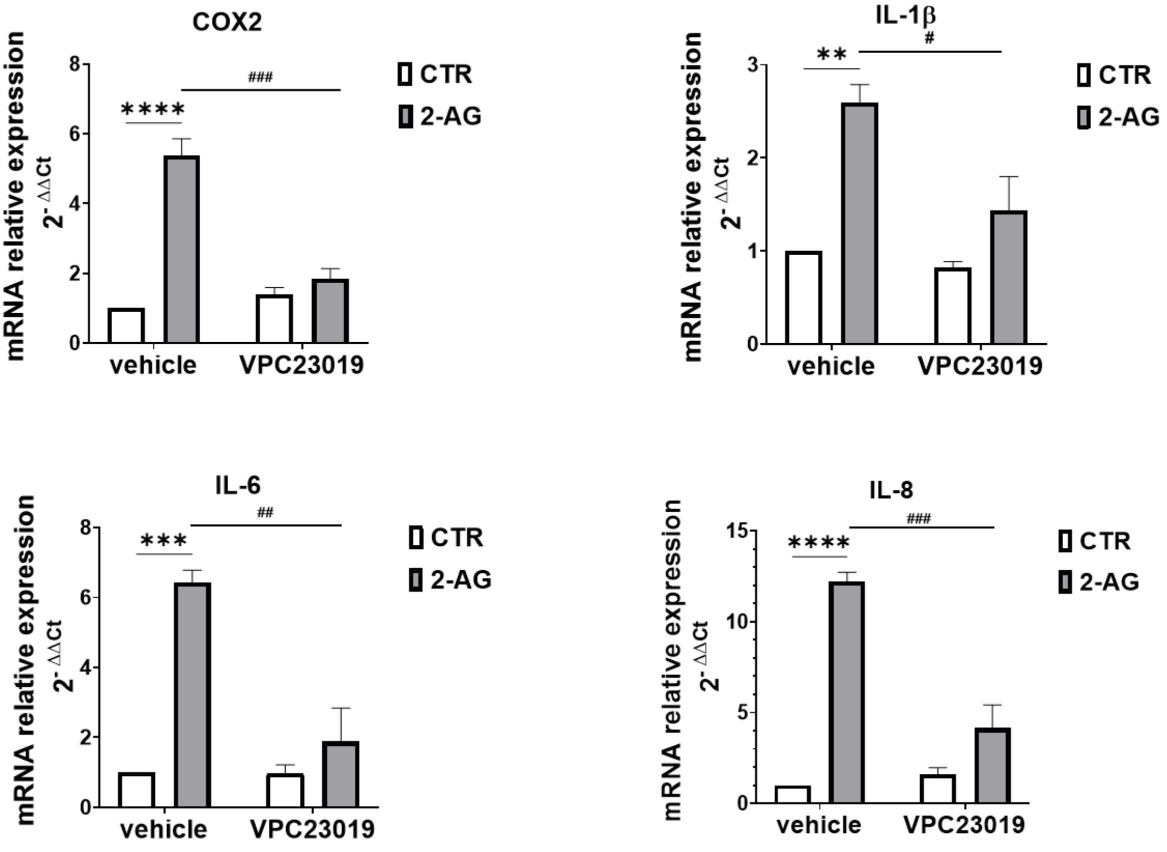

Figure S3. 2-AG pro-inflammatory action relies on $S1P_3$. Serum-starved endometriotic epithelial cells were pretreated or not with the $S1P_1/S1P_3$ antagonist VPC23019 (10 μM) for 45min before being challenged with 10 μM 2-AG for 24 h. mRNA quantitative analysis of COX2, IL-1β, IL-6 and IL-8 was performed by qPCR. Results, analyzed with the $2^{-\Delta\Delta Ct}$ method, were obtained using β-Actin as a housekeeping gene and individual inflammatory factors of the unchallenged specimen as a reference gene. The effect of VPC23019 on 2-AG-induced inflammatory effect was statistically significant by two-way ANOVA followed by Bonferroni's post hoc test (#$p < 0.05$; ##$p < 0.01$; ###$p < 0.001$).

A B

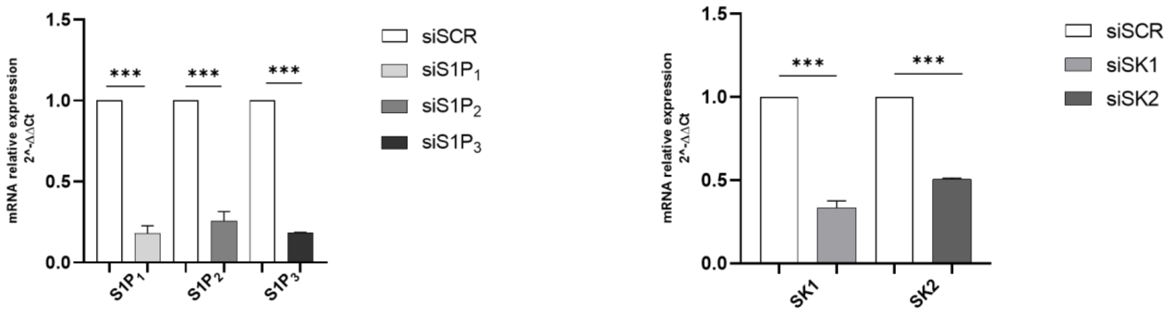

Figure S4: Silencing efficiency of S1PR and SKs in endometriotic epithelial cells. (**A**) qPCR analysis was performed in endometriotic epithelial cells transfected with nonspecific siRNA (SCR) or with siRNA specific for $S1P_1$ or $S1P_2$ or $S1P_3$. Results, analyzed with the $2^{-\Delta\Delta Ct}$ method, were obtained using β-Actin as a housekeeping gene and each receptor subtype in cell transfected with siSCR used as calibrator. Data are mean ± SEM of three independent experiments performed in triplicate. The effect of $S1P_1$- $S1P_2$- or $S1P_3$ -siRNA transfection is statistically significant by Student's t-test ***$p < 0.001$. (**B**) qPCR analysis was performed in endometriotic epithelial cells transfected with nonspecific siRNA (SCR) or with siRNA specific for SK1 or SK2. Results, analyzed with the $2^{-\Delta\Delta Ct}$ method, were obtained using β-Actin as a housekeeping gene and each receptor subtype in cell transfected with siSCR used as calibrator. Data are mean ± SEM of three independent experiments performed in triplicate. The effect of SK1- or SK2-siRNA transfection is statistically significant by Student's t-test ***$p < 0.001$.
